# Assessment of depth perception with a comprehensive disparity defined letter test: A pilot study

**DOI:** 10.1371/journal.pone.0271881

**Published:** 2022-08-12

**Authors:** Wei Hau Lew, Daniel R. Coates

**Affiliations:** University of Houston College of Optometry, Houston, Texas, United States of America; Cairo University Kasr Alainy Faculty of Medicine, EGYPT

## Abstract

Current clinical tests mostly assess stereopsis with crossed disparity at near. These tests are designed with fine targets (high spatial frequency) and may fail to capture the “functional stereopsis” in real-world scenes, which consist of a range of spatial frequencies (SFs). We developed a stereo letter test that can assess crossed and uncrossed stereoacuity at near and far, at different SFs defined by the letter size. The test consists of disparity-defined letters embedded in random-dot stereograms. At each letter size, the letters are arranged in sets of trigrams like in the Pelli-Robson chart. The letter sizes correspond to SFs ranging from 0.3 to 2cpd. Within each triplet, all letters have the same disparity and the amount of disparity decreases after each set. Subjects report the letters verbally to determine the smallest disparity at each letter size. Twenty-four subjects were tested with eight different charts: crossed vs. uncrossed disparity at far and near, with two versions (different letter sequences). The disparity sensitivity function (DSF) had an inverted U-shape, with decreasing sensitivity for smaller stereo letters. The subjects had better stereopsis at far than near. All the subjects had lower stereo thresholds with crossed disparity than uncrossed, consistently at both distances. We found no effect of age or heterophoria on the DSF. The charts have good test-retest reliability (Pearson’s r = 0.89, p<0.001) and are easy to perform. Our results with stereo letters as stimuli are comparable to results from studies using depth corrugations. This stereo acuity letter test permits assessment of stereopsis at different testing distances, directionality of disparity, and across a range of SFs, which can help diagnose selective stereo losses in binocular vision anomalies and monovision. Assessment of stereopsis at different SFs may provide additional information for understanding daily stereovision demands than the conventional tests.

## Introduction

Stereopsis, or the ability to perceive depth, is vital to estimate distance during navigation, prevent falls, and support accurate motor reactions, and fine hand movements. Depth can only be judged when both eyes function optimally, providing slightly different horizontal views of the same scene from the interpupillary distance [1]. Random dots stereograms (RDS) which have no monocular cues are typically used to assess global stereopsis [2].

Good and balanced image quality between the two eyes is essential for stereopsis. Image degradation by uncorrected refractive error or ocular disorders such as amblyopia will impair depth perception. Amblyopes have been shown to have selective deficit in contrast sensitivity function mainly in the high spatial frequencies (SF) range corresponding to the loss of visual acuity [3]. However, most clinical tests are designed with small and fixed target sizes, measuring stereopsis only at high SF. As a result, most amblyopes are diagnosed as “stereoblind” when they fail these tests [4]. It is unclear if the stereo deficits in amblyopia occur across the entire spectrum of SFs, or only at high SFs, similar to their contrast sensitivity function. Natural visual scenes consist of a wide range of SFs. Measuring stereopsis with fine targets (high SFs) may not reflect the complete picture of the underlying stereo visual function. Moreover, the visual system is more sensitive in the intermediate range of SF [5]. For example, the Pelli Robson contrast chart was designed to assess middle SFs targets, and has been proposed to be more relevant to real-world vision and a better indicator of Quality of Life (QoL) than visual acuity. Similarly, a stereo test that can determine stereopsis at a range of SFs provides more holistic insight into the impact of stereo deficiency on visual-motor functions such as hand-eye coordination, balance skills, and reading speed [6, 7].

**Fig 1:**
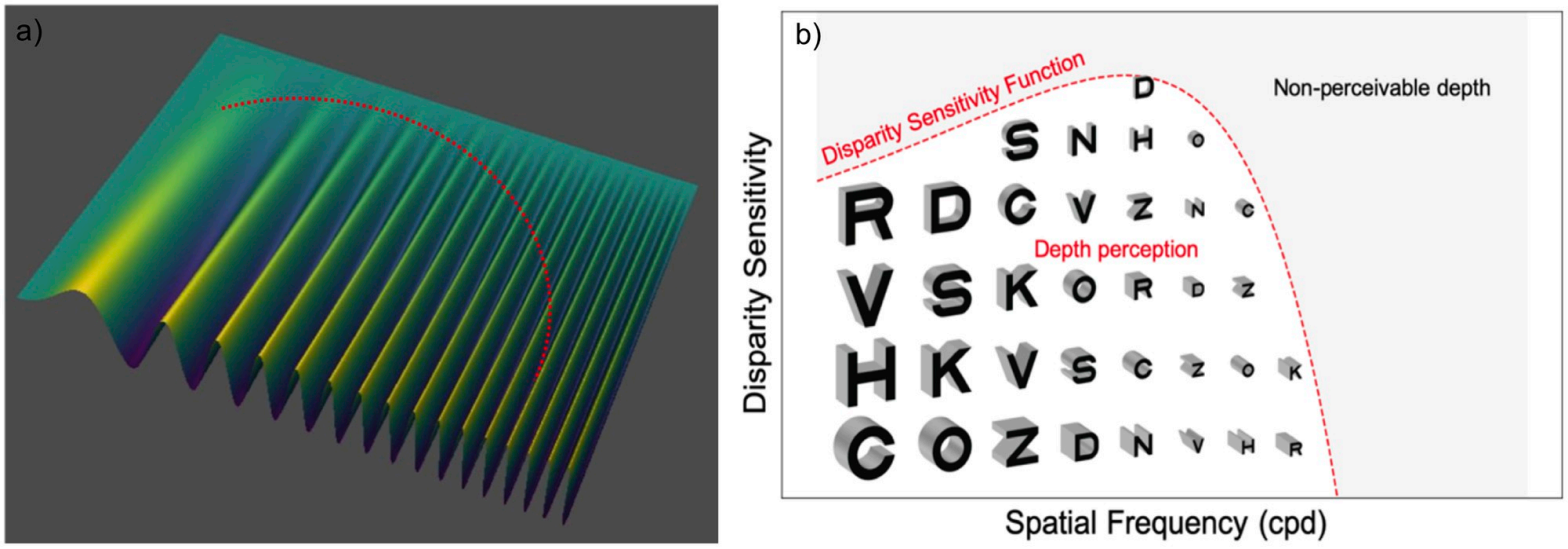
Depth corrugations as a function of spatial frequency. a) Illustration of a surface propagating in sinusoidal depth used in other studies. The dotted red curve depicts the disparity sensitivity function, DSF with peak sensitivity at middle spatial frequency. b) Illustration of letter optotypes in various depth and sizes used in our disparity defined letter test to assess DSF. The dotted red curve indicates the hypothetical stereo threshold at each spatial frequency (or letter size). The shaded region outside of the DSF is the non-perceivable depth region.

Sinusoidal corrugations in depth made using RDS have been used to study the mechanisms behind stereo processing and disparity tuning channels, as shown in Fig 1a [8–12]. The depth corrugation thresholds of spatial frequencies are generally an inverted U-shape, analogous to the contrast sensitivity function (CSF). This curve, also known as the disparity sensitivity function (DSF), is shifted to the left by ten-fold compared to the CSF, with a peak at approximately 1cpd. Besides understanding the basic mechanism of stereo processing, measuring the DSF has great potential as a diagnostic test to evaluate the selective losses of stereopsis at a particular SF and assess stereovision demand in the real world consisting of a spectrum of SFs. However, these psychophysics studies are constrained to laboratory settings.

Apart from the fine targets, most clinical tests only assess crossed disparity (front) at near. Only a few distance stereo tests are commercially available [13]. Assessment of distance stereopsis is needed for certain occupational tests, sports vision, and in diagnosing and management of divergence excess -intermittent exotropia [14, 15] which may have stereo deficits only at far that would be overlooked if clinicians only test near stereopsis.

There are two types of disparity directions: crossed (front) and uncrossed (back). The mechanism behind uncrossed disparity is still poorly understood. It has been reported that some individuals may show anisotropy in the disparity directionality [16, 17]. Studying this anisotropy would help us to understand the different processing mechanisms in these two types of disparity. In addition to horizontal disparity, stereopsis can also be achieved with vertical disparity. Vertical disparity provides information on size, curvature, slant, and layout in visual space [18]. Since small-angle vertical misalignment often presents along with horizontal strabismus, assessment of vertical disparity may provide insights into vertical vergence.

The purpose of this study is to translate laboratory-based depth corrugation tests for a clinical setting with simple letter optotypes, as illustrated in Fig 1b. We have included other parameters to address the limitations of currently available clinical tests. We developed and piloted a disparity defined letter test that can assess crossed and uncrossed stereo acuity near and far, with different letter sizes corresponding to different spatial frequencies.

## Materials and methods

This study is divided into three experiments. Five subjects participated in Experiment 1, twenty-six in Experiment 2, and six subjects from Experiment 2 sat for additional experimental conditions in Experiment 3. All the subjects were recruited from the optometry department; either students, faculty, or staff members who had eye examinations and self-reported no ocular disorders. All subjects were screened with an inclusion criteria of monocular visual acuity of at least 20/25 for both far and near, and stereo acuity of <40 arcsecs tested with the Randot Stereo Test. Subjects wore their habitual correction during the experiments. Subjects with presbyopia wore their reading glass during the near chart conditions. Basic binocular vision assessments such as cover test and ocular motility test were performed prior to the experiment. Dissociated phoria for far and near were tested with Maddox rod and Modified Thorington Charts. Written informed consent was obtained from all participants before the experiment, and the experimental procedures were approved by the Institutional Review Board of the University of Houston. All experimental procedures were performed under this protocol.

### Experiment 1: Pilot study for disparity sensitivity function with letter optotypes

**Fig 2:**
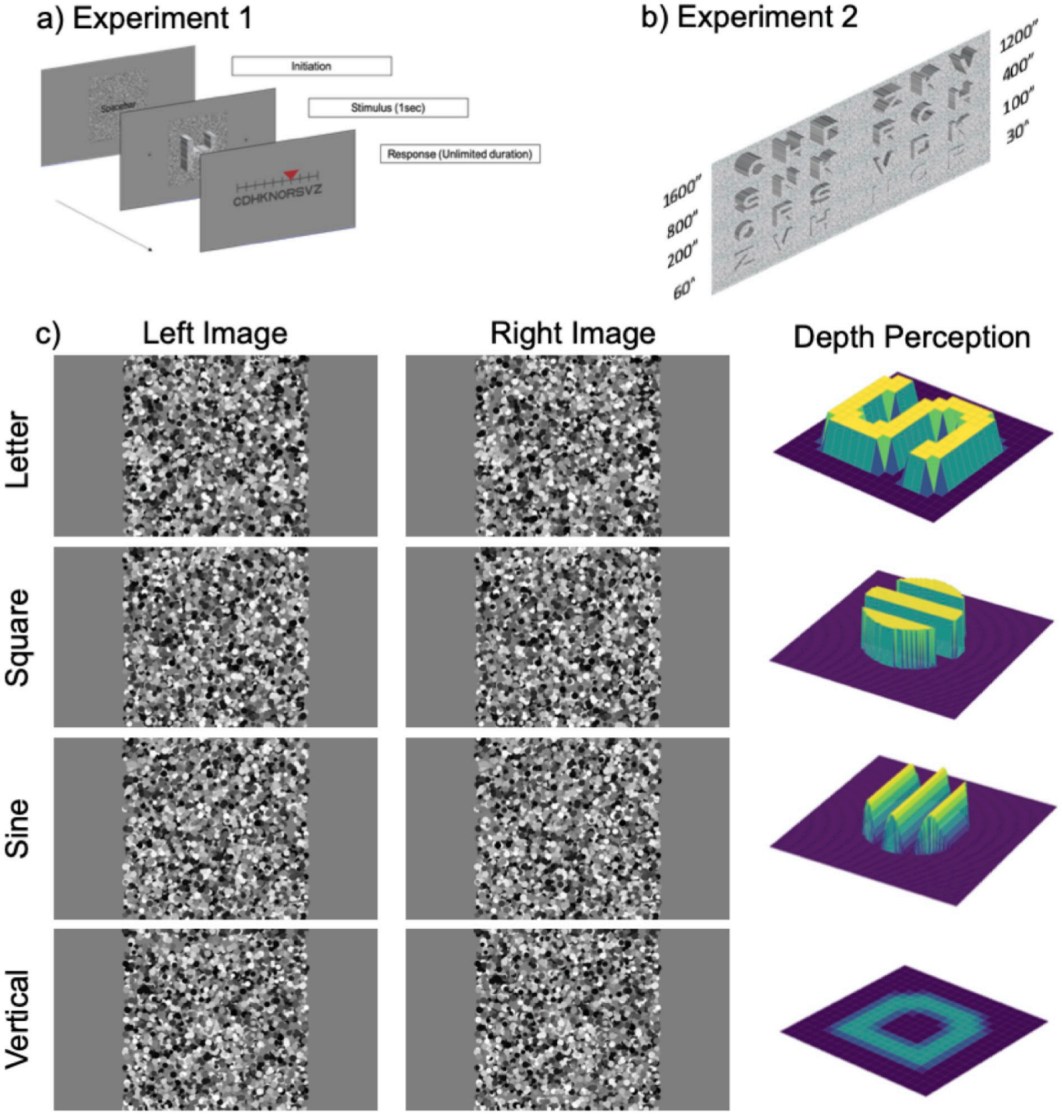
Experimental set-up and stimulus. a) Sequence of stimulus presentation in Experiment 1. The single letter appears in depth embedded in Random Dot Stereogram for 1 sec, followed by a response page. Subjects used the arrow keys to select the letter seen in the previous slide. b) Illustration of the near stereo letter chart in Experiment 2. Each page corresponds to one spatial frequency or letter size. On each page, the letters are presented in trigrams (four rows and two columns). Within each triplet, all letters have the same disparity with a total of eight disparity steps tested. The disparity steps, in arc seconds, were listed next to each set of trigrams (not shown during the experiment). Subjects read the letters aloud from left to right starting from the top row. c) Example of stereo stimuli used in Experiment 2 and 3. By fusing the left and right images, readers can see a letter “S” appearing in depth (crossed disparity or front) as illustrated in the rightmost column. In Experiment 3, we included square and sinewave depth corrugations in a circular patch. The sine gratings have disparity gradient, while square gratings have an “all or none” disparity profile. In the last row, readers can appreciate the letter “D” presented with a vertical disparity. Letters with vertical disparity do not appear to “pop-out” or “cave-in,” but rather have a shimmering luster appearance.

Custom programs written in Python with the PsychoPy library [19] were used to generate Random Dot Stereograms (RDS) for all the experiments. RDS are more sensitive in detecting stereo deficits in amblyopia and contain minimal monocular cues [20, 21]. In the RDS, Sloan letters appeared in depth (closer than the display plane as illustrated in Fig 2c top row). The task was to identify the letter embedded in the RDS. Since our goal is to replace gratings with letter optotypes for future clinical translation, we first piloted the data in five subjects with normal stereovision to determine if the DSF curve is the same with letters target instead of sinusoidal depth corrugations [22]. We performed the task with a haploscope system with two high pixel density miniature LCD monitors (Feelworld Master MA5 5-inch, 1920 x 1080 pix). In the monitors, a Sloan letter (C, D, H, K, N, O, R, S, V, or Z) appeared in depth, embedded in the RDS with a total of 11,400 dots, as shown in Fig 2a. The monitors were placed at 115cm away from the observer, and the size of each dot was between 3 to 9 pix (random), corresponding to a visual angle of 30 to 90 arc secs. To generate a crossed disparity image, a portion of the dots seen by the left eye were displaced horizontally to the right while corresponding dots seen by the right eye had similar offsets but in the opposite direction. The amount of horizontal displacement defined the amount of disparity. The disparity thresholds were estimated from a 1-Up & 3-Down staircase method with seven reversal points. At a fixed letter size the test started with a moderate disparity (~300”) and reduced with three consecutive correct responses; otherwise, it increased with a wrong response. Each letter was presented for 1sec, followed by a response page. On the response page, all ten Sloan letters were shown on a slider, and the subject used the left and right arrow keys to select the letter they perceived in depth. The letters were presented at seven different sizes corresponding to spatial frequencies of 0.25, 0.5, 1, 1.5, 2, 2.5, and 3.75 cycles per degree (cpd) in individual blocks after a training session. The order of blocks was randomized and repeated twice to determine the mean disparity threshold at each spatial frequency. For the two lowest spatial frequencies, the monitors were moved to 29cm away from the observer, without rescaling of the dot density or sizes.

### Experiment 2: Development and assessment of far and near stereo letter charts with method of constant stimuli with a larger sample size

To reduce the testing duration from the adaptive staircases in Experiment 1, we modified the stereo charts for combinations of near (40cm) and far (3m) and crossed, uncrossed disparity with two versions (different letter sequence) with method of constant stimuli. Stereo presentation was achieved with Nvidia active shutter goggles (flicker rate = 60Hz) and an Asus VG248QE 3D monitor (1920 x 1080 pix, 24 inches). The near charts comprised six pages, with the letters on each page organized into triplets (4 rows x 2 columns, for a total of 8 sets of triplets), similar to the Pelli-Robson chart [23], as illustrated in Fig 2b. Within each triplet, all letters had the same disparity. The letters were presented with disparities of 1600, 1200, 800, 400, 200, 100, 60, and 30 arcsecs. The letter sizes corresponded to SFs ranging from 0.3 to 2cpd, with center-to-center separation of 1.5x of the letter size and 1.3x between each line. Each dot was randomly drawn between 4–6 pix diameter (each pix subtends a visual angle of 2.2 arcmin) with random luminance between black and white. The dot density for the RDS is 100 dots per degree^2^ with average luminance of ~40cd/m^2^ at 40cm through the active shutter goggles with surrounding room lighting. The far charts (3m) were scaled to match the near charts. Only one letter was presented on the screen in the far chart, and had the same sequence as the near charts, as illustrated in Fig 2c top row. The dot size and density were scaled accordingly to match the distance chart with average luminance of ~30 cd/m^2^. Subjects reported the letters verbally to determine the smallest disparity that could be read until two errors were made within one triplet. Each subject was tested with eight different charts: crossed (front) vs. uncrossed (back) disparity at far and near, with two versions (different letter sequences). We recruited twenty-six subjects with normal stereovision from the age group of 20 to 50-year-old. The sequence of the experimental conditions was randomly assigned to each subject.

### Experiment 3: Comparison to depth corrugation gratings and vertical disparity

Experiment 3 is an extended apparatus and stimuli of Experiment 2 with additional conditions. In Experiment 3a, the letters were presented with vertical disparity instead of horizontal disparity. The letters seen by the right eye were shifted upwards while the left letters were shifted in the opposite direction. With vertical disparity, the letters do not appear as “pop-out” or “cave-in” but rather had a shimmering appearance as depicted in Fig 2c last row. We tested at near and far with two different versions.

Sloan letters have a disparity profile similar to modulation of a square grating (“all-or-none” disparity profile), with an equivalent spatial frequency of 5 strokes per letter. On the contrary, sinusoidal depth corrugations (smooth disparity gradient) are commonly used in other studies. To compare our results to the prior literature [22, 24, 25], we tested with two different horizontal disparity profiles: square and sinewave corrugations with minimum zero disparity on the reference plane. The targets were presented with 2.5 cycles, corresponding to five strokes in a letter, in a circular patch, as illustrated in Fig 2c second and third row. The patch size is matched to the heights of the letters tested in Experiment 2. Subjects verbally reported the orientation of the disparity-defined gratings. Subjects were trained to identify the orientations relative to the midline before the experiment: vertical (0°), horizontal (90°), left tilt (-23°), far left (-67°), right tilt (23°), and far right (67°).

In Experiment 2, the entire letter size changes with spatial frequency, with smaller letter sizes for higher spatial frequencies. Typically, targets such as depth corrugations will retain the same size target at different spatial frequencies. To test whether the size is a confounding factor, we included an additional condition with square-wave corrugations in a fixed overall patch size. Instead of reducing the patch size, or visual angle to define the spatial frequency, more number of cycles were added for higher SFs condition in this condition. The patch sizes were 8 degrees for the near charts, equivalent to the largest target size at 0.3cpd, and 5 degrees for the distance chart for the target size at 0.5cpd. Similarly, subjects reported the orientation of the patch. The set-up, dot size, and dot density were the same as Experiment 2.

### Data analysis

**Fig 3:**
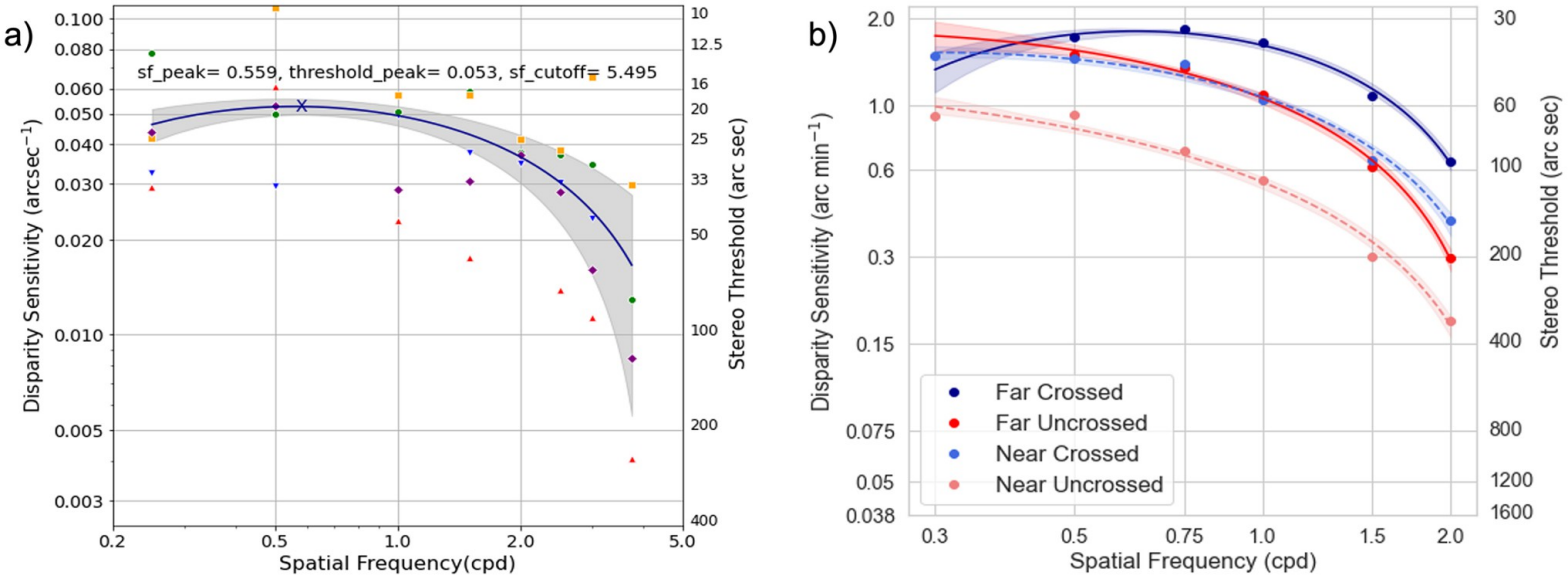
Disparity sensitivity function curve with letter charts. a) DSF curve from the pilot study in Experiment 1 using a staircase method on a haploscope. The blue line represents the log parabola quadratic fit from the results of five subjects, while the color markers are individual results. The DSF curve with letter stimuli had a U-shape, in accordance with results from sinusoidal gratings in the literature. The dark blue ‘X” indicates the estimated peak of disparity sensitivity. b) The DSF curve for near, far, crossed, and uncrossed conditions in Experiment 2 using Method of Constant Stimuli. The far DSF curve (solid lines) is higher than the near conditions, indicating a superior stereopsis at far than near. The DSF curve for the crossed disparity is also higher than uncrossed disparity for both far and near, implying higher sensitivity for crossed disparity (front). The 95% confidence bounds, shaded regions were calculated with bootstrap resampling (1000 repeats).

We performed data analyses using Python and the Numpy/Scipy scientific libraries [26]. We used the non-parametric Wilcoxon test and Friedman test to compare the result between the different conditions. A p-value of 0.05 is the threshold for statistical significance. Pearson correlations were used to compare the test-retest repeatability between the two charts. To determine the confidence intervals for the log-parabola quadratic fit used to model the results, bootstrap resampling was used [27, 28]. Specifically, the empirical data for each condition were resampled (with replacement) to generate a new simulated dataset. This dataset was then refitted to obtain an additional estimate of the parameters. This procedure was repeated for 1000 times to build a distribution of parameter fits. These distributions are reported in Table 1 and were used to determine the shaded 95% confidence intervals plotted in Figs 3, 6 and 7.

**Table 1:** 

Condition	Spatial Frequency Peak (cpd), x	Sensitivity Peak (arcmins^-1^), y	Stereo Threshold (arcsecs)
Far Crossed (Letters)	0.627 [0.570, 0.684]	1.806 [1.765, 1.847]	33.2
Far Uncrossed (Letters)	0.202 [0.057, 0.347]	1.784 [1.349, 2.219]	33.6
Near Crossed (Letters)	0.301 [0.230, 0.372]	1.525 [1.447, 1.603]	39.3
Near Uncrossed (Letters)	0.060 [0, 0.149]	1.208 [0.605, 1.811]	49.7
Far Vertical (Letters)	0.305 [0.066, 0.544]	0.483 [0.356, 0.610]	124.2
Near Vertical (Letters)	0.480 [0.445, 0.515]	0.354 [0.345, 0.363]	169.5
Far Square Corrugations	0.322 [0.284, 0.360]	2.178 [2.134, 2.222]	27.5
Near Square Corrugations	0.352 [0.296, 0.406]	1.500 [1.447, 1.553]	40.0
Far Sinusoidal Corrugations	0.484 [0.429, 0.539]	2.059 [2.034, 2.084]	29.1
Near Sinusoidal Corrugations	0.248 [0.185, 0.311]	1.500 [1.407, 1.593]	40.0
Far Fixed Size Square Corrugations	0.680 [0.656, 0.704]	2.030 [2.012, 2.048]	29.6
Near Fixed Size Square Corrugations	0.348 [0.280, 0.416]	1.336 [1.279, 1.393]	44.9

List of the coordinates of the maximal points of the log parabola quadratic fit for each condition and estimated stereo threshold in arcsecs. The x-coordinate for vertex position of the curve is indicated by the spatial frequency peak (cpd) while the y-coordinate corresponds to the sensitivity peak, in arcmins. Larger spatial frequency peak value, x means that the inverted U-shape curve is shifted to the right. Higher sensitivity peak value, y means the curve is shifted upwards with better stereo sensitivity. The 95% confidence intervals are listed in the brackets.

## Results

### Experiment 1

Five subjects (Mean age = 27.2±2.95 year old, 2 female) participated in this experiment. The stereo threshold for each spatial frequency was estimated from the mean of the last six reversal points in each staircase. The stereo thresholds, in arcsecs, were then converted into disparity sensitivity by calculating the reciprocal value. We averaged the result across subjects and fit the data with a simple log parabola-quadratic fit, for comparison to other studies [9, 11]. The estimated peak (x-value, sf_peak) of the curve is at 0.557**±**0.078 cpd with 0.053**±**0.002 arcsec^-1^ sensitivity (y-value). This peak sensitivity is equivalent to ~18 arc secs. The curve intersected the x-axis at a cut-off frequency (sf_cutoff) of ~5.5cpd when extrapolated, as shown in Fig 3a. The DSF curve obtained with letter stimuli in our pilot experiment has the same inverted U-shape results from sinusoidal gratings in other studies. Since there will be different disparity sensitivity values for each SF for each condition, the results for each condition can be represented as parameters in a quadratic fit for easier interpretation. The subsequent data will be presented in terms of the location (x and y coordinate) of the peak of the inverted U plot to represent the best disparity sensitivity, and the relative position (either horizontal or vertical displacement) from baseline result. The x and y coordinate of the peak sensitivity of the plot can be used for direct comparison to other studies.

### Experiment 2

Twenty-six subjects participated in this experiment but two subjects were excluded from the analysis due to stereo deficit and unable to perform the task. The mean age of the subjects was 33.6±8.12 years (13 female). We averaged the stereo thresholds across the subjects and the two repeats (two versions of the charts). The stereo thresholds, in arcsecs, were then converted into disparity sensitivity by calculating the reciprocal value. The data for each condition were fitted with the log parabola-quadratic with bootstrap resampling (1000 repeats) to determine 95% confidence intervals. Similar to the result in Experiment 1, we found that the disparity-defined letter sensitivity function had a U-shaped curve in all the other conditions, as shown in Fig 3b. A higher DSF plot indicates higher sensitivity and better stereopsis and vice versa.

#### A) Comparison between near and far disparity thresholds

Most subjects had higher sensitivity or lower stereo threshold at far than near (Wilcoxon Signed Rank test, T = 377.5, p<0.001). On average, distance stereo acuity for crossed disparity reached up to ~35 arcsecs (at 0.75cpd), while the average near threshold was ~46 arcsecs (at 0.5cpd) at the peak of the curve with crossed disparity. The near DSF curve is shifted vertically downwards compared to the far condition (solid vs. dashed dark blue lines for the crossed condition and solid vs dashed red line for the uncrossed condition). The circle markers in Fig 3b represent the mean value across the subjects for each condition at different spatial frequencies. In the far conditions (solid lines), the lowest spatial frequency tested was 0.5cpd and the fit extrapolated the data to 0.3cpd, resulting in a larger 95% confidence interval. Table 1 summarizes the coordinate for the maximal turning points of the inverted U-plot for each conditions. The spatial frequency peak denotes the x-coordinate, while the sensitivity peak denotes the y-coordinate. From the table, the sensitivity peak values (third column) for the far conditions are significantly larger than for the near condition, indicating a more superior stereo sensitivity at far.

#### B) Comparison between crossed and uncrossed

All the subjects had higher sensitivity and lower stereo thresholds with crossed disparity than uncrossed, consistently in both distances (Near condition p<0.001, Far condition p<0.001). In Fig 3b, the solid and dashed red curves (uncrossed conditions) are lower than the solid and dashed blue curves, respectively. Based on the fit and Table 1, the curve for the uncrossed is shifted to the left and lower than the crossed conditions. In other words, the subjects were able to identify the letters that “pop-out” more easily than those that “cave in”.

#### C) Comparison between the two versions of the letter chart

**Fig 4:**
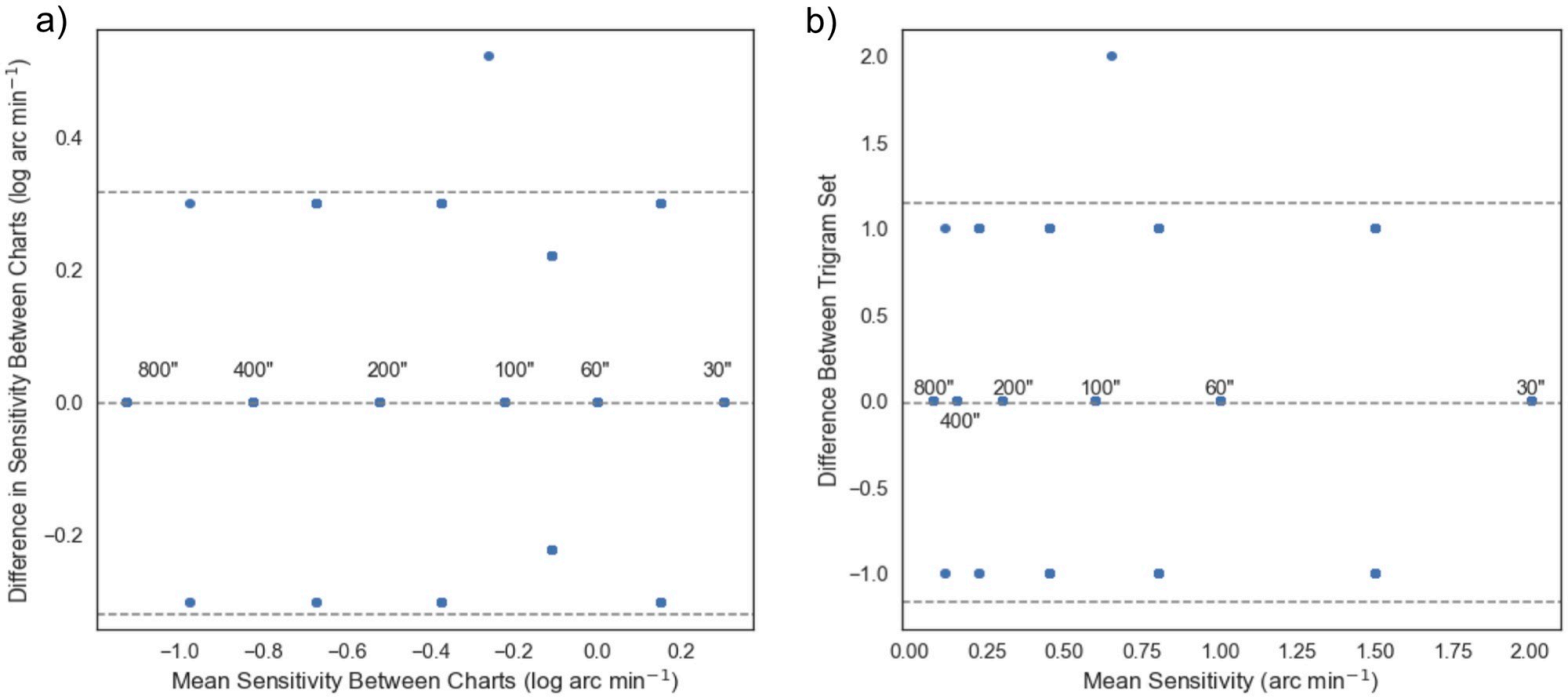
Bland-Altman plot for test-retest repeatability between the two versions of charts. a) Most results are within a 95% confidence interval, denoted by the horizontal dashed lines. Note that the units used in x and y abscissa are in log value. For easier reference, the values in arcsecs are labeled next to the points. The two charts have good test-retest repeatability. b) To test on the repeatability within neighboring trigram set, we transformed the result, in arc minutes, into trigrams sequence, 1 to 8 (1600 arc secs for 1 and 8 for 30 arc secs). The test-retest repeatability is within 1 set of trigrams between the two charts. In other words, the individual’s performance was in the range of one neighboring set of trigrams between the two trials. Note that the units here are in linear scale.

We also compared the results for the two chart versions, 1 and 2. The subjects had good test-retest repeatability within the two versions of the chart (Pearson’s r = 0.89, p<0.001). Fig 4a shows the difference vs. mean stereo threshold across the two charts. Only one out of the 528 stereo thresholds recorded were outside of the 95% confidence interval, as shown by the single outliers above the dashed lines. It is worth pointing out the unequal disparity steps between the trigrams that we used in the design to match other clinical tests. To investigate if the result is consistent within the neighboring set of trigrams, we relabeled the trigrams into a sequence score, from 1 to 8, with 1 for the maximum disparity, 1600” while a score of 8 for the minimum disparity, 30”. With discrete sequence score, we found that the standard deviation between the two charts was close to 1 neighboring set of trigrams, as shown in Fig 4b. In other words, the charts had good test-retest repeatability, with a difference of only one disparity step, or one set of trigrams apart between the two repeats.

#### D) Age factor

To investigate whether age was a factor in the results, we divided the subjects into three age groups, by decade. Within the twenty-four subjects, nine were in their 20s, eight were in their 30s, and seven were in their 40s. An additional analysis using Kruskal-Wallis test was conducted to examine the differences in sensitivity according to the age groups. No significant differences (Chi square = 0.222, p-value = 0.89) were found across the different age groups. In our study, subjects in the early presbyope group performed similarly to the younger age group with the appropriate near correction.

#### E) Correlation to phoria

**Fig 5:**
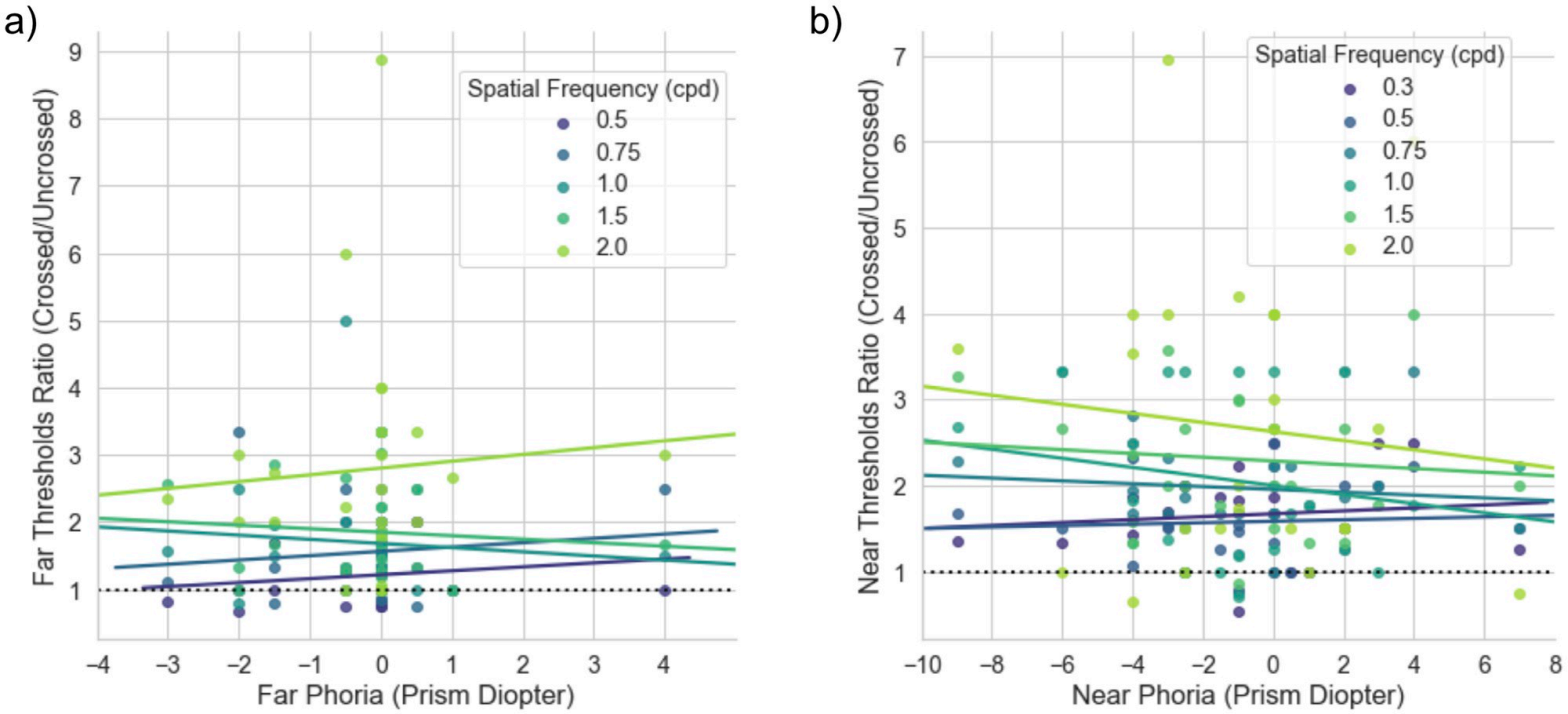
Scatterplots of the ratio of crossed/uncrossed disparity as a function of heterophoria. A negative value on the x-axis indicates the exophoria in prism diopters while a positive value for esophoria. A ratio of >1 indicates superior sensitivity to the crossed disparity. The different color hues represent the ratio at different spatial frequencies. a) Result for far phoria. We do not see any correlation between far phoria to directional bias. b) Result for the near phoria. Similarly, we do not see any correlation pattern between near phoria and directional bias. The horizontal dotted line indicates the ratio of 1:1, where stereo threshold for crossed and uncrossed disparity is equivalent. Note that the scales on the axes are different between the two plots.

On average, the crossed disparity has a lower threshold than the uncrossed disparity, we wanted to test if the bias is dependent on the nature of an individual’s heterophoria; e.g. esophoria more bias towards crossed disparity vs exophoria for the uncrossed disparity. To investigate the effect of heterophoria, scatterplots of the stereo threshold ratio of crossed relative to uncrossed disparity as a function of their phoria were computed. This bias may also be SF-dependent, therefore we plotted different hues for each spatial frequency. In Fig 5, a negative value in the x-axis denotes exophoria while a positive value indicates esophoria. The dotted line at y = 1 is when stereo threshold for the crossed disparity is the same as uncrossed. All the slopes at each spatial frequency, are above the y = 1 line and are not significantly different from a flat line, implying that the directionality of disparity sensitivity (crossed or uncrossed) does not correlate to one’s heterophoria in this group of participants. A correlation between directionality of phoria and would result in a slant but we do not see this pattern in Fig 5.

### Experiment 3

Six subjects from Experiment 2 participated in this experiment with additional conditions for comparison to the literature.

#### A) Vertical disparity of letter chart

**Fig 6:**
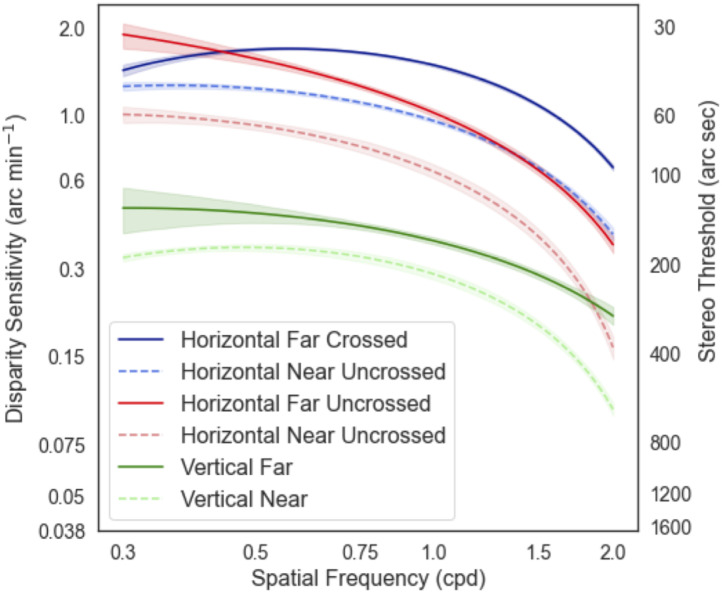
Disparity Sensitivity Function (DSF) for the vertical disparity. The red and blue solid and dashed lines are the results from six subjects for crossed and uncrossed horizontal disparity at near and far. The solid green line is the DSF curve for vertical disparity at far while the dashed green line is for the near condition. The vertical DSFs were significantly lower. The subjects were less sensitive to vertical than horizontal disparity. The shaded region denotes the 95% confidence interval from bootstrap resampling (1000 repeats).

Compared to the horizontal disparity (crossed and uncrossed disparity), the stereo thresholds were significantly higher for vertical disparity for both distances. The vertical DSF curve was shifted vertically downwards and flatter, as shown by the green lines in Fig 6. A non-parametric Friedman test of differences among repeated measures at far, between the crossed, uncrossed, and vertical disparity conditions were conducted and rendered a Chi-square value of 105.72, which was significant (p<0.001). Similarly, it was also statistically significant at the near condition; the Chi-square value was 123.44 with a p-value <0.001.

#### B) Comparison between letters with square and sine wave depth corrugations

**Fig 7:**
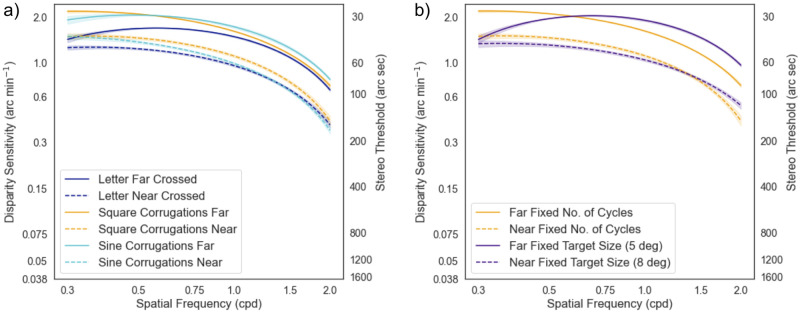
Comparison between stereo letter and gratings corrugations. a) Stereo sensitivity with letter targets were lower than square and sine corrugations at the low spatial frequencies range. The sensitivity at high spatial frequencies is comparable between letters and gratings corrugations. b) The DSF curves between the fixed target size and fixed number of cycles with square wave corrugations are relatively similar. However, at high SFs, the sensitivity was slightly better when a greater number of cycles were added in a fixed target size. The shaded region denotes the 95% confidence interval from bootstrap resampling (1000 repeats).

The Friedman test across the different stimuli (letter vs. square vs. sine gratings) was conducted, and the Chi-square value was 24.75 which was significant (p<0.001) for far testing distance, indicating a difference in stereo sensitivity between the three conditions. Fig 7a shows that the sensitivity was higher for square and sine wave corrugations at low spatial frequencies, but this discrepancy diminishes at high spatial frequencies. On the other hand, the Chi-square value was 4.129 which was not significant (p = 0.127) for near.

#### C) Comparison between fixed target size vs. fixed number of cycles

To investigate if the size is a confounding factor, we compared two different types of square wave corrugations at the same spatial frequency: either a fixed number of cycles or the overall stimulus size. We compared the result in Experiment 3B to 3D. We found at near they are comparable (T = 74, p = 0.615). However, at far they were barely significant (T = 74, p = 0.04). We noticed a trend that the DSF curve for fixed size is flatter at the higher SFs, as shown in Fig 7b. In other words, a fixed large target size with a greater number of cycles may improve the sensitivity at higher spatial frequencies.

## Discussion

From a series of experiments, we found that the disparity sensitivity function tested with stereo letters varying in size had a similar inverted U-shape as the disparity sensitivity function measured with depth corrugations reported previously. The charts showed good test-retest reliability. Most of the repeated measurements were within one neighboring disparity step. Our participants had better stereopsis at far than near with crossed than uncrossed disparity. However, we did not find any correlation to age or phoria.

Previous studies have reported conflicting results between stereo thresholds and viewing distances. Some reported no correlation between stereo threshold and testing distances [29], while others found a more superior stereopsis at far [30]. Nonetheless, these results vary with the types of tests. Bradshaw & Glennester has found that the thresholds were elevated at the closest testing distance (28.5cm) with depth corrugation stimuli [31]. Our result is in agreement with their findings that distance stereoscopic vision is better as compared to near. The DSF curve is slightly shifted upwards in the far condition. Poorer near stereopsis may be attributed to a higher possibility of vergence error. The visual system has better control of smaller vergence angles at far, meanwhile a higher probability of over or under convergence at near fixation. Another possible explanation is that near stereopsis has a higher potential of mismatched accommodation and vergence, while at distance, depth perception is mainly driven by vergence only. Having a distance stereopsis can be advantageous to assess depth perception if one fails the near stereo test, especially in individuals with amblyopia.

Mixed results were reported in the literature regarding the anisotropy of crossed and uncrossed disparity. While most have found higher sensitivity with crossed disparity [32–34], some found the opposite effect [35] or no significant difference [36]. Our results showed a more superior sensitivity in crossed disparity than uncrossed. The DSF curve for the uncrossed disparity is vertically lower than the crossed disparity. This anisotropy may be attributed to multiple explanations. Generally, our visual system is more experienced in detecting objects in front of the fixation plane than those behind the fixation. Moreover, sensitivity to crossed disparities has also been found to develop earlier than uncrossed [37] and the range of fusional vergence is larger for convergence than divergence [38]. Neuroimaging and electrophysiological studies have also reported this anisotropy in evoked potentials amplitude, waveform, and the processing site [34, 39]. The fit from Table 1 shows that the peak of the DSF curve is not symmetric between the crossed and uncrossed conditions. The uncrossed DSF curve is to the left, suggesting perhaps there are different sets of tuning channels between them, which is consistent with the hypothesis of two neural pools of disparity detectors, one for crossed and another for uncrossed disparity detection [40].

Shippman & Cohen have reported that individuals with esophoria have higher sensitivity with uncrossed disparity and vice versa for the exo type due to asymmetry in Panum’s fusional areas (16). However, we do not find any correlation between the phoria and directionality bias. This may be attributed to the smaller number of subjects in our study, especially in the esophoria group. In addition to that, we do not know where the subjects were fixating during the experiment. It could be eso or exo fixation.

This current study is focused on piloting the stereo letter charts hence we only included individuals with good visual acuity and stereopsis. The results are reliable within the 20 to 50-year-old group, despite the onset of presbyopia. However, we acknowledge the decline of stereopsis in the aging population [41] and would be an important aspect to consider on its own in our future work.

To the best of our knowledge, our study is the first to measure the DSF with vertical disparity. The vertical disparity curve has a lowpass shape, similar to the horizontal disparity, but shifted downwards by three-fold and flatter. Unlike horizontal disparity, vertical disparity does not provide information about the absolute or relative depths from the fixation plane. It induces vertical vergence which can be a useful clinical tool in the diagnostic and management of vertical and paralytic strabismus. Panum’s Fusional Area which is larger in the horizontal than vertical dimension may account for the horizontal-vertical anisotropy. Electrophysiological studies in animals have also shown that binocular neurons are more broadly tuned to horizontal disparity than vertical [42].

To compare the letter DSF results to other studies, a series of controlled experiments were conducted. The disparity profile of the letter chart is similar to a square-wave corrugation. The “ink area” of a letter is “elevated” from the zero-disparity fixation plane, as shown in Fig 1c top row. However, most of the stimuli used in other studies were sinusoidal depth corrugations with a smooth disparity gradient. Conversely, a square-wave corrugation has an abrupt change in disparity and may affect the stereo discrimination ability. Hence, square and sinusoidal gratings were included for comparison. However, the stereo sensitivity was better at the low SF range with both square and sinusoidal corrugations as compared to letter optotypes. We postulate that with large letters (low SF), one needs to pay attention to the entire target while with square or sinusoidal corrugations, one can distinguish the orientation by looking at parts of the target.

In this study, spatial frequency is quantified as a function of letter sizes. The sizes of the letters could be another confounding factor. To study the effect of size, square grating were compared with two control conditions: a fixed size with a varying number of cycles versus a fixed number of cycles but varying overall size at the same spatial frequency. In the former condition, the number of cycles in a patch increases at higher SFs, while in the latter, the number of cycles was fixed at 2.5, but the overall patch size decreases with higher SFs. We hypothesize that more information can be obtained with more cycles in a fixed size, thus improving the stereo sensitivity, especially at high SFs as seen in our result. Related results have also been reported in the contrast sensitivity literature, where more cycles in a patch will reduce the threshold [10]. A larger target size also provides a larger visual search area for horizontal eye movement which is essential for depth perception. Although decreasing the overall size with a fixed number of cycles limits the “aperture size,” it provides a fair comparison, especially the information derived from spatial frequency. In addition to that, the Panum’s fusional area also scale according to the target size, or in our case, the letter size. Having a large area of gratings corrugations may affect the Panum’s Fusional area differently. In summary, measuring DSF with letters of varied sizes may have discrepancies in the lowest or highest SFs compared to gratings.

However, recognition of disparity defined letters make the task easier, faster, and more clinically relevant. Grating corrugations are affected by the horizontal vs. vertical corrugation anisotropy. Studies have shown that the visual system is more sensitive to horizontal depth corrugation than vertical [11, 43, 44]. Letters, on the other hand, contain different orientations and curves to balance out the anisotropy. The tasks are different; one is letter recognition while the other requires orientation discrimination. Letter charts also have lower guessing rate compared to the orientation discrimination in the gratings charts.

We acknowledge that the DSF curve may differ slightly between letters and gratings corrugations. Similar discrepancy has been observed in contrast sensitivity between the two different types of targets. Dioptric blur, or uncorrected refractive error reduces sensitivity substantially in letters compared to gratings [45]. There are also some uncertainties regarding the specific spatial frequencies involved in letter recognition [46] which potentially complicates the theoretical interpretation of underlying mechanisms of the letter DSF. We compared our result to the normative dataset (61 subjects) from Reynaud & Hess 2015 [22]. Despite different fitting methods, our log parabola-quadratic fit found almost identical results to the truncated log-parabola model used in their qDSF with sinusoidal depth corrugations. Our letter DSF curve has almost the same stereo sensitivity as shown in their Fig 4b.

Despite the consistent shape of the DSF curve across the experiments, it is worth noting that the position of the curve depends on the experimental factors such as basic elements of the random dot: dot density [42], size [10], and contrast luminance. For example, the DSF curve for Experiment 1 peaks higher than Experiment 2. This may be attributed to the higher dot density achieved from the high pixel monitors and the staircase method. Ideally, we want a moderate density, too low and too high will reduce the stereo performance. To achieve higher density, the overall size of the display must be small and compact. The monitor used in Experiment 2 has the standard display resolution therefore we are unable to achieve denser dots without dropping any frames. The density we used in Experiment 2 can roughly cover all the surface area of the letters. On the other hand, larger dots prevent testing smaller letters. The combination of dot density and dot size we chose were close to the limit without compromising the frames. Since stereoacuity is best with higher contrast [47, 48], the DSF curve also follows the similar pattern. In Experiment 1, we also tested with lower contrast as controlled condition. We found that the DSF curve shifted vertically downwards as contrast reduces.

Our study has several limitations as follows: 1) in the distance charts, only one letter was presented at a time while all twenty-four letters were presented simultaneously in the near chart. The surrounding letters in the near chart may cause distraction, confusion, and crowding effects. On the other hand, presenting one letter at a time (like in the far chart) increases the testing time significantly and may cause fatigue. 2) the lowest disparity tested was 30 arcsecs. Adding a lower stereo threshold such as 20 or 15 arcsecs may reveal a higher peak sensitivity in the DSF. Adding another disparity step will increase the duration and difficulty level for some observers with a higher threshold. This test is designed to be a screening test for observers with binocular vision anomalies; hence we have included coarse disparity steps similar to other clinical tests for easy comparison across the tests. 3) a larger sample size or inclusion of a subset of stereo impaired individuals in a future study would be useful.

## Conclusion

We have developed a new stereo acuity test that can assess stereopsis at different distances, directionality of disparity and across a range of SFs. The test has the potential to assess the selective stereo loss in binocular vision anomalies and provides more information than conventional tests for understanding daily stereovision demands.
